# Supplementary material for: Empirical methods for controlling false positives and estimating confidence in ChIP-Seq peaks
Source: BMC Bioinformatics. 2008 Dec 5;9:523. doi: 10.1186/1471-2105-9-523 (PMC2628906; doi:10.1186/1471-2105-9-523)
Supplement: Additional File 1 — A variety of html documents from the USeq web site detailing the available applications, their best usage, output file type descriptions, command line menus, etc. [file 1471-2105-9-523-S1.zip › USeqUserGuides/JavaDoc.zip!/JavaDoc/trans/tpmap/class-use/TPMapFeature.html]

Uses of Class trans.tpmap.TPMapFeature


|  |  |  |
| --- | --- | --- |
| **Overview** \| **Package** \| **Class** \| **Use** \| **Tree** \| **Deprecated** \| **Index** \| **Help** | |  |
| PREV   NEXT | **FRAMES**    **NO FRAMES**     **All Classes** |  |


---


## **Uses of Class trans.tpmap.TPMapFeature**

| Packages that use TPMapFeature | |
| --- | --- |
| **trans.tpmap** | For maping, filtering, and calculating statistics about text version bpmap files. |

Uses of TPMapFeature in trans.tpmap

| Methods in trans.tpmap with parameters of type TPMapFeature | |
| --- | --- |
| `static boolean` | `TPMapDuplicateFilter.matches(TPMapFeature a, TPMapFeature b)`             Only checks coordinates! |

---


|  |  |  |
| --- | --- | --- |
| **Overview** \| **Package** \| **Class** \| **Use** \| **Tree** \| **Deprecated** \| **Index** \| **Help** | |  |
| PREV   NEXT | **FRAMES**    **NO FRAMES**     **All Classes** |  |


---
